# Hacking hematopoiesis – emerging tools for examining variant effects

**DOI:** 10.1242/dmm.049857

**Published:** 2023-02-24

**Authors:** Michael Gundry, Vijay G. Sankaran

**Affiliations:** ^1^ Division of Hematology/Oncology, Boston Children's Hospital, Harvard Medical School, Boston, MA 02115, USA; ^2^ Department of Pediatric Oncology, Dana-Farber Cancer Institute, Harvard Medical School, Boston, MA 02115, USA; ^3^ Broad Institute of MIT and Harvard, Cambridge, MA 02142, USA

## Abstract

Hematopoiesis is a continuous process of blood and immune cell production. It is orchestrated by thousands of gene products that respond to extracellular signals by guiding cell fate decisions to meet the needs of the organism. Although much of our knowledge of this process comes from work in model systems, we have learned a great deal from studies on human genetic variation. Considerable insight has emerged from studies on presumed monogenic blood disorders, which continue to provide key insights into the mechanisms critical for hematopoiesis. Furthermore, the emergence of large-scale biobanks and cohorts has uncovered thousands of genomic loci associated with blood cell traits and diseases. Some of these blood cell trait-associated loci act as modifiers of what were once thought to be monogenic blood diseases. However, most of these loci await functional validation. Here, we discuss the validation bottleneck and emerging methods to more effectively connect variant to function. In particular, we highlight recent innovations in genome editing, which have paved the path forward for high-throughput functional assessment of loci. Finally, we discuss existing barriers to progress, including challenges in manipulating the genomes of primary hematopoietic cells.

## Teaser

**Summary:** Thousands of genomic loci have been linked to hematopoietic traits and diseases, yet many await functional validation. Here, we discuss recent advances in genome editing and challenges in using these techniques to assess variant function in primary hematopoietic cells.

## Introduction

“The life of the flesh is in the blood” (Leviticus 17:11). Every second, our body produces more than 2 million red blood cells to help deliver oxygen to our tissues, more than 1 million platelets to help maintain hemostasis, and more than 1 million white blood cells to enable our immune function. This production process, termed hematopoiesis, is highly organized, and responds to extracellular signals to prioritize production of specific lineages in periods of injury, stress or acute illness. Chronic deficiencies in the production line or defects within any of the individual products may lead to pathological consequences to the individual.

**Fig. 1.**
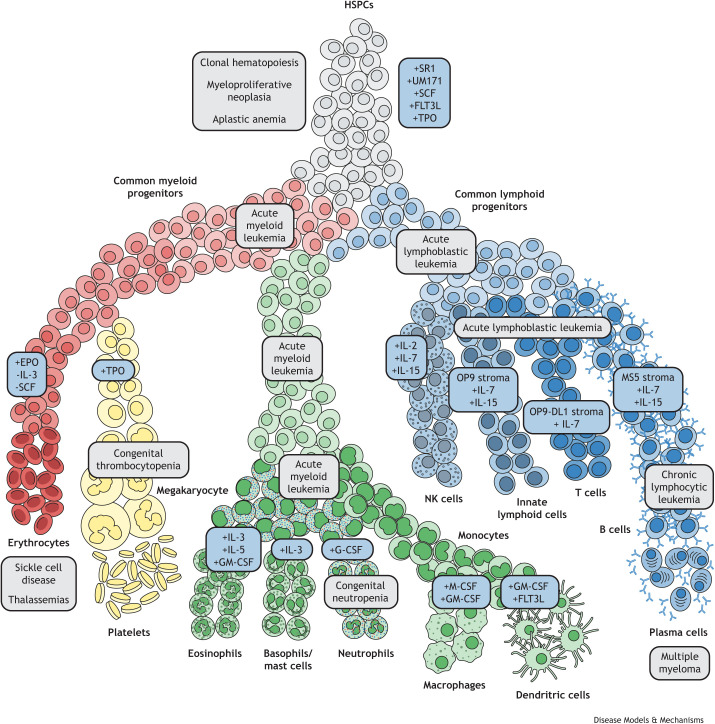
**Flow diagram of hematopoiesis.** Schematic depicting the cellular trajectories of hematopoiesis. Disease processes affecting individual cell types at different stages of differentiation are highlighted in gray boxes. The combined use of small molecules (SR1 and UM171) and human cytokines (SCF, FLT3L and TPO) can expand hematopoietic stem and progenitor cell (HSPC) populations (top blue box) (Fares et al., 2014). Cytokines used to differentiate HSPCs into erythrocytes (Giani et al., 2016), platelets (Perdomo et al., 2017), eosinophils (Shalit et al., 1995), basophils/mast cells (Kepley et al., 1998), neutrophils (Rao et al., 2021), macrophages (Way et al., 2009), dendritic cells (Swartz and Nair, 2022), natural killer (NK) cells (Spanholtz et al., 2010), innate lymphoid cells (Hernández et al., 2021), T cells (Singh et al., 2019) and B cells (Dubois et al., 2020; Kraus et al., 2014; Luo et al., 2009; Spanholtz et al., 2010; Hernández et al., 2021) are highlighted in blue boxes. EPO, erythropoietin; FLT3L, FMS-related tyrosine kinase 3 ligand; G-CSF, granulocyte colony-stimulating factor; GM-CSF, granulocyte macrophage colony-stimulating factor; IL-2, interleukin-2; IL-3, interleukin-3; IL-5, interleukin-5; IL-7, interleukin-7; IL-15, interleukin-15; M-CSF, macrophage colony-stimulating factor; SCF, stem cell factor; SR1, StemRegenin 1; TPO, thrombopoietin.

Our understanding of the organization of the hematopoietic system and the mechanisms governing its operation are rooted in the hypothesis that it is a hierarchical process with all blood elements derived from a common precursor cell (Maximow, 1909) (Fig. 1). Since the discovery of the ‘polyblast’, now termed the hematopoietic stem cell (HSC), studies in mice and other model organisms have helped characterize factors responsible for the maintenance of HSCs and the differentiation process through which mature blood cells are formed (Alexander et al., 1996; Liggett and Sankaran, 2020). Although these studies have helped us understand this process at a high level, there are clearly aspects of hematopoiesis that are unique to humans and insights that can emerge through the study of human genetic variation (Medetgul-Ernar and Davis, 2022). The completion of the initial draft of the human genome and subsequent advances in sequencing technologies have helped accelerate the pace of discovery (Shendure et al., 2017). And, more recently, the establishment of large-scale biobanks and cohorts linking genomic variation and clinical data, such as the Trans-Omics for Precision Medicine (TOPMed) (Taliun et al., 2021) and the UK Biobank (Bycroft et al., 2018), have provided the framework and data for increasingly well-powered genome-wide association studies (GWASs) and sequencing-based rare variant association studies. In the past few years alone, thousands of new germline genetic loci have been described that contribute to variation in various hematopoietic traits or increase the risk for different blood diseases, including blood and immune cell phenotypes (Chen et al., 2020; Vuckovic et al., 2020), clonal hematopoiesis (see Glossary, Box 1) (Bick et al., 2020; Brown et al., 2022 preprint; Kar et al., 2022) and blood cancers (Bao et al., 2020; Mitchell et al., 2016; Vijayakrishnan et al., 2019). As the number of individuals in these databases grows, our ability to identify the genetic contribution to hematopoietic phenotypes and diseases will grow in tandem. However, until these variants are experimentally validated, and the underlying biological mechanisms are uncovered, there are limited insights to inform therapeutic and preventive strategies. Additionally, with increasing sequencing of patients with blood diseases, particularly those thought to arise due to monogenic causes, the growing list of variants of unknown significance and unknown mechanism represents a major bottleneck in human genetics. Although the global scientific community is growing, an increase in manpower is not enough to address the chasm between the exponentially growing identification of both monogenic and polygenic variation in disease, and our mechanistic insights. It is paramount that tools for experimental validation of putative causal variants keep pace with variant discovery. Somewhat fortuitously, a new generation of tools for high-throughput variant assessment has emerged, bolstered by the recent development of next-generation genome-editing technologies. Here, we discuss traditional and emerging experimental approaches that address the variant-to-function problem for blood phenotypes and diseases.

“Somewhat fortuitously, a new generation of tools for high-throughput variant assessment has emerged, bolstered by the recent development of next-generation genome-editing technologies.”

**Box 1. Glossary**

**Acute lymphoblastic leukemia:** a cancer of the blood and bone marrow affecting lymphoid progenitors, including immature T cells, B cells and natural killer (NK) cells.

**Aldolase A:** an enzyme involved in the fourth step of glycolysis and found predominantly in red blood cells and muscle tissue. Deficiency results in dysregulation of energy homeostasis within red blood cells, leading to membrane instability and rupture (see ‘hemolytic anemia’).

**Alpha-thalassemia:** an inherited hemoglobinopathy characterized by insufficient production of alpha-globin chains due to large deletions at the alpha-globin locus or point mutations in hemoglobin subunit alpha 1 (*HBA1*) and/or hemoglobin subunit alpha 2 (*HBA2*). Affected individuals have chronic anemia and are often dependent on blood transfusions.

**B-cell lymphoma/leukemia 11A (BCL11A):** a transcription factor involved in the regulation of gene expression at the beta-globin locus. In the postnatal period, BCL11A orchestrates the switch from high transcriptional activity at hemoglobin subunit gamma 1/2 (*HBG1/2*) loci to high transcriptional activity at the hemoglobin subunit beta (*HBB*) locus through changes in chromatin looping and enhancer–promoter interactions. An erythroid-specific enhancer of BCL11A located in an intron of the *BCL11A* gene is currently the target of multiple gene therapy trials aimed at restoring high levels of fetal hemoglobin to ameliorate disease in beta-hemoglobinopathies (see ‘fetal hemoglobin’ and ‘sickle cell disease’).

**Beta-thalassemia:** an inherited hemoglobinopathy characterized by insufficient production of beta-globin chains due to large deletions at the beta-globin locus or point mutations in *HBB*. Affected individuals have chronic anemia and are often dependent on blood transfusions.

**Beta-2 microglobin (B2M):** a component of major histocompatibility complex (MHC) class I molecules that present intracellular peptide fragments to cytotoxic CD8^+^ T cells. Loss of B2M leads to near-complete loss of surface expression of MHC class I. Ablation of *B2M* is currently being used in a number of clinical trials to create ‘off the shelf’ CAR-T therapies that are resistant to allorejection.

**C-C chemokine receptor type 5 (CCR5):** a G-protein-coupled receptor on the surface of T lymphocytes, macrophages and immature dendritic cells that regulates trafficking and effector functions. It also acts as a co-receptor for membrane fusion and viral entry of human immunodeficiency virus (HIV) viral particles. Individuals with homozygous loss-of-function mutations in CCR5 are resistant to HIV infection. It is currently a target of gene-editing therapies for prevention and amelioration of HIV infection.

**Clonal hematopoiesis:** an age-related disorder characterized by the emergence of a detectable population of blood cells that share somatically acquired mutation(s). It is a risk factor for the development of leukemia and atherosclerotic cardiovascular disease.

**Diamond-Blackfan anemia:** a congenital disorder of the bone marrow characterized by ineffective production of red blood cell progenitors. It is a genetically heterogenous disorder enriched for mutations in ribosomal protein genes that cause aberrant translation of key erythroid maturation factors.

**DNA methyltransferase 3A (DNMT3A):** a *de novo* DNA methyltransferase responsible for deposition of methyl groups on the C-5 carbon of cytosines in DNA. In humans, the enzyme preferentially methylates cytosines at CG dinucleotides, which are enriched at gene promoters. Loss-of-function mutations in *DNMT3A* are among the most common somatic mutations found in clonal hematopoiesis and hematopoietic malignancies, likely acting through aberrant epigenetic programs interfering with normal hematopoiesis.

**Fetal hemoglobin:** the predominant oxygen carrier protein during gestation and in the perinatal period. It is composed of two alpha-globin subunits (α_2_) encoded by *HBA1/2* and two gamma-globin subunits (γ_2_) encoded by *HBG1/2.* After birth, BCL11A orchestrates an epigenetic reprogramming of the beta-globin locus to increase HBB expression at the expense of HBG1/2, resulting in the transition from fetal hemoglobin to adult hemoglobin. Individuals with sickle cell disease who have higher levels of fetal hemoglobin have less-severe symptoms, and drugs that increase baseline fetal hemoglobin levels represent the most effective disease-modifying agents for patients with sickle cell disease.

**Hemolytic anemia:** a form of anemia caused by increased breakdown of red blood cells either in the blood stream (intravascular) or in other organ systems (extravascular). It can present in patients as an acquired or inherited disorder. Inherited forms include defects in red blood cell membranes (membranopathies), such as hereditary spherocytosis, or defects in red blood cell metabolism, such as aldolase A deficiency (see ‘aldolase A’).

**Immune dysregulation:** a maladaptive process through which normal immune system functions are corrupted, leading to autoimmune disease, cancer and hyperinflammatory states. Patients often have co-existing immunodeficiencies.

**Landing pad:** a synthetic or endogenous segment of DNA that can be used for precise and efficient genomic integration of one or more genetic elements.

**Myeloid neoplasia:** a group of malignant disorders specifically affecting the myeloid lineage of the hematopoietic system. Examples include acute myeloid leukemia, myelodysplastic syndrome and myeloproliferative neoplasia.

**RNA-binding motif protein 38 (RBM38):** an RNA-binding protein that regulates alternative splicing during terminal erythropoiesis. Individuals with inherited variants in *RBM38* are at increased risk for the development of anemia.

**Sickle cell disease:** a group of inherited hemoglobinopathies characterized by aberrant polymerization of hemoglobin secondary to missense mutations in *HBB*. Affected individuals have chronic anemia, vaso-occlusive episodes (pain crisis) and increased infection susceptibility.

**Terminal erythropoiesis:** the process by which nucleated red blood cell progenitor cells undergo maturation into anuclear erythrocytes (red blood cells).

## Computational approaches for variant prioritization and interpretation

The validation of genetic variants has long been hampered by difficulty in identifying the appropriate biological context for testing and the absence of high-throughput experimental approaches. This has led to significant investment into the development of computational tools for variant prioritization and mechanistic inference. One particularly valuable approach is fine-mapping, which leverages population genetics and linkage disequilibrium patterns to identify likely causal variants and also can be integrated with epigenomic annotations to help discern putative mechanisms at specific loci (Schaid et al., 2018).

Variant interpretation and prioritization tools often employ evolutionary conservation or variation constraint to estimate the pathogenicity of a variant (Adzhubei et al., 2010; Vaser et al., 2016). These tools are often more sensitive in coding regions, in which variant impact on coding sequence is more readily discerned, but this can also be applied to non-coding elements. Nonetheless, interpretation of non-coding variants is challenging, as regulatory elements are less conserved across species, and defining the consequences of these changes can be harder. Multiple collaborative efforts, such as the Encyclopedia of DNA Elements (ENCODE) project (ENCODE Project Consortium, 2004), the Roadmap Epigenomics project (Bernstein et al., 2010) and the International Human Epigenome Consortium (Stunnenberg et al., 2016), have helped address this problem through the generation of genome-wide epigenomic maps across diverse human tissues. These epigenomic maps label tissue-specific regulatory elements, which are enriched in causal variants for blood and immune phenotypes and can be used to prioritize GWAS hits for downstream functional validation (Cano-Gamez and Trynka, 2020). Integrating these epigenomic maps into variant prediction algorithms, such as Combined Annotation-Dependent Depletion (CADD) (Kircher et al., 2014), has led to improved accuracy in prioritizing functional and pathogenic variants. Emerging data on tissue-specific three-dimensional genome interactions should further improve variant prioritization efforts. For instance, Javierre et al. (2016) applied promoter capture Hi-C, which detects interactions between gene promoters and *cis*-regulatory elements, to 17 human primary hematopoietic cell types and computationally linked thousands of previously uncharacterized GWAS single-nucleotide polymorphisms to their putative target genes.

Early epigenomic datasets were derived from mixed populations of cells masking important cell-type- or state-specific regulatory elements. Incorporating single-cell omics data into variant interpretation algorithms has led to improved sensitivity to detect causal variants driving blood cell traits (Ulirsch et al., 2019) and autoimmune diseases (Zhang et al., 2021). Unfortunately, the high sparsity and signal dropout in many single-cell datasets limits the power to accurately detect colocalization of a variant and an epigenomic state. To overcome this problem in sparsity and noise, our laboratory, alongside collaborators, recently developed an approach termed Single Cell Analysis of Variant Enrichment through Network propagation of GEnomic data (SCAVENGE) (Yu et al., 2022), which uses network propagation to better discern phenotype-relevant cells in sparse single-cell data and map trait- and disease-relevant genetic variation to the appropriate cellular context. Specifically, SCAVENGE allowed us to link variants associated with severe coronavirus disease (COVID-19) to immature CD14^+^ monocyte populations and map the dynamic changes of acute lymphoblastic leukemia (Box 1) risk predisposition along the B-cell developmental trajectory. These examples highlight the particular relevance of SCAVENGE to studying the influence of genetic variation on blood cells, as both phenotypes were restricted to rare hematopoietic populations with subtle global transcriptional differences compared to their neighbors that were missed in prior bulk and single-cell analyses.

In the coming years, the emergence of high-resolution single-cell datatypes and datasets (Regev et al., 2017; van der Wijst et al., 2020), as well as the development of new computational tools, will undoubtedly improve our ability to fine-map and prioritize variants. With the increasing availability and affordability of whole-genome sequencing, it is inevitable that most individuals living in the developed world will soon have the opportunity to have their genomes sequenced, and some individuals will harbor variants that increase their risk of various hematopoietic disorders, such as clonal hematopoiesis and blood cancers. Ideally, this genetic information could be used clinically to guide more frequent screening or lifestyle modifications to reduce disease risk. However, to properly inform these clinical recommendations, we must experimentally recapitulate the phenotypic consequences of these genetic variants (MacArthur et al., 2014), which is a tall task given the growing list of putative variants.

## Harnessing genetic tools to hack hematopoiesis

Congratulations, you have your list of putative causal variants. Now an important and formidable challenge in validation emerges. The most straightforward approach for validation of a variant associated with blood phenotypes/diseases is to directly investigate the gene product at the RNA or protein level using blood or bone marrow cells from individuals harboring these variants (Wolfe et al., 1982). However, this presumes that appropriate samples are available and that the impact gene has already been implicated in the phenotype/disease state. Most variants identified from GWASs do not meet these criteria and, often, even variants identified through sequencing approaches of rare patients and cohorts may not as well. Instead, investigators have classically relied upon molecular cloning and transfection/transduction of the gene of interest into cell lines (Box 2).

**Box 2. Traditional approaches for validating genetic variants**

Through exogenous delivery of complementary DNA (cDNA), investigators have identified variant-specific defects in transcription, splicing, protein stability and protein/cellular function. This approach can be applied in a high-throughput manner through delivery of cDNA pools containing all possible variants in a gene (Coyote-Maestas et al., 2022; Majithia et al., 2016; Melnikov et al., 2014; Mighell et al., 2018). In theory, these saturation mutagenesis screens could be performed for every human gene, essentially solving the variant-to-function problem for coding variants. However, many coding sequences are too large to clone/transfect, and delivery of an exogenous product often leads to supraphysiologic expression, confounding the biologic interpretation. Furthermore, these high-throughput approaches have been difficult to apply to primary cells, precluding the ability to properly assess the effect of variants on cellular phenotype, particularly in the context of physiologic differentiation or transient cell states.

For non-coding variants, the most common classic validation approach has been to use reporter assays in which the regulatory element encompassing the variant is placed upstream of a minimal promoter and a reporter gene, often GFP or luciferase. The success of such assays has led to multiple high-throughput versions that incorporate nucleic acid barcodes and allow for thousands of putative variants/regulatory elements to be assayed in parallel. Aside from a few studies in primary T-cells (Bourges et al., 2020; Mouri et al., 2022), these massively parallel reporter assays (MPRAs) have been performed using cell lines. Although MPRAs are effective at identifying variants that abrogate the activity of strong enhancers, the minimal genomic regions profiled precludes the identification of variants with more complicated effects on three-dimensional genome structure (Inoue et al., 2017).

The use of exogenous assays for functional validation has proven extremely useful in identifying causative variants and facilitating a deeper exploration of the biology of specific hematopoietic cell types. Exogenous delivery of complementary DNA has led to the characterization of promoter variants affecting transcription in beta-thalassemia (Box 1) (Orkin et al., 1983) and splicing variants driving alpha-thalassemia (Box 1) (Felber et al., 1982), and has also been used to identify thermolabile variants in aldolase A (Box 1) causing hemolytic anemia (Box 1) (Kishi et al., 1987). More recently, systematic profiling of hundreds of variants in DNA methyltransferase 3A (*DNMT3A*; Box 1), a key driver of clonal hematopoiesis and myeloid neoplasia (Box 1), identified a key factor regulating DNMT3A turnover (Huang et al., 2022). Massively parallel reporter assays (MPRAs) have also been used to successfully validate putative non-coding variants linked to various blood/immune cell traits, including the identification of an enhancer variant causing downregulation of RNA-binding motif protein 38 (RBM38; Box 1) and a subsequent defect in terminal erythropoiesis (Box 1) (Ulirsch et al., 2016). Unfortunately, the validation rate of putative non-coding variants using MPRAs has been low (Tewhey et al., 2016), likely due to the non-physiological background in which the experiments were performed.

Investigators interested in using MPRAs to screen a set of putative non-coding variants should plan their experiments carefully to mitigate the shortcomings of the approach. These assays can be optimized for use in more physiologically relevant cell types, such as primary hematopoietic stem and progenitor cells (HSPCs), or run in the setting of a physiologically relevant perturbation, such as addition of a stress signal or infectious agent. Alternatively, a landing pad (Box 1) could be used to drop in the reporter construct at a specific genomic locus to mitigate cell-to-cell variation in reporter expression based on the local chromatin milieu (Durrant et al., 2022). However, the prudent approach would be to transition to an endogenous system for the validation of both coding and non-coding variants (Box 3).

**Box 3. A Cas-cade of new technologies**

Since tools were developed for homologous recombination (Thomas and Capecchi, 1987), endogenous gene targeting has become the gold standard for assessing the effect of mutations on cellular and organismal function. The low efficiency of homologous recombination placed a high cost and time burden on the approach, effectively preventing it from being applied in a high-throughput fashion. The discovery of RNA interference (RNAi) (Lee et al., 1993) and the subsequent hacking of the endogenous silencing machinery using custom small interfering RNA (siRNA)/short hairpin RNA (shRNA) molecules (Fire et al., 1998) created a cost-effective tool for the systematic disruption of endogenous gene activity. The approach is amenable to multiplexing through the creation of siRNA libraries (Moffat et al., 2006). However, its utility is limited to gene knockdown/knockout, and there can be frequent off target effects (Jackson and Linsley, 2010). Therefore, these approaches have recently been used less frequently since the discovery and adoption of the CRISPR/Cas9 system and derived screening tools by the research community (Deltcheva et al., 2011; Jinek et al., 2012).

**Fig. 2.**
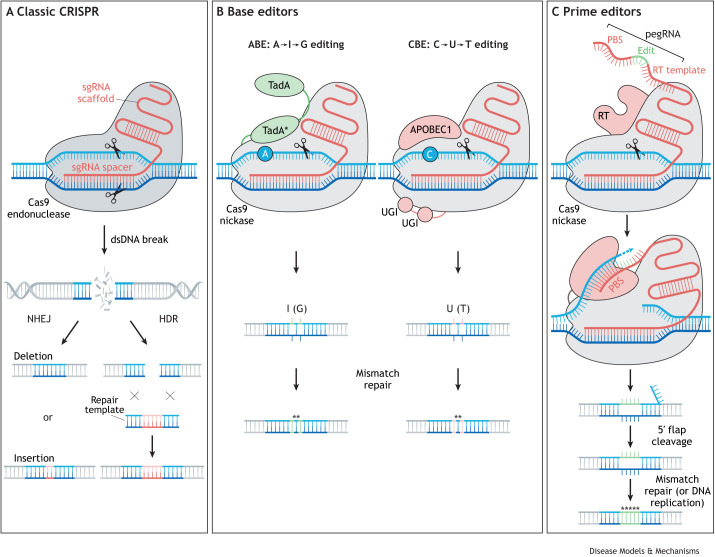
**CRISPR/Cas9-based tools and delivery approaches.** (A) Cas9 endonucleases form complexes with single-guide RNAs (sgRNA) by binding to the constant scaffold region. The Cas9/sgRNA complex then scans the genome until it identifies a region with complementary DNA to the spacer sequence (first 20 bp of sgRNA). Transient binding of the sgRNA to its cognate target DNA facilitates the formation of a double-strand DNA (dsDNA) break. Repeat cycles of cleavage and repair with non-homologous end joining (NHEJ) eventually leads to mis-repair through the addition or removal of nucleotides (left arrow). By delivering an alternative homologous template for repair, one can introduce specific edits at the target site through homology-directed repair (HDR; right arrow). (B) Base editing avoids the formation of dsDNA breaks by using a mutant Cas9 ‘nickase’, which is only capable of creating a single-stranded nick. Adenine base editors (ABE; left) consist of a Cas9 nickase fused to two copies of tRNA adenine deaminase (TadA), one of which (TadA*) is mutated to accept DNA as a substrate, leading to deamination of adenines to inosines (treated as guanine by DNA polymerase). Cytosine base editors (CBE; right) are composed of Cas9 nickase fused to APOBEC1, a cytidine deaminase that converts cytidine to uracil (treated as thymine by DNA polymerase). The addition of two uracil-DNA glycosylase inhibitor (UGI) domains to the fusion protein prevents the conversion of uracil back to cytidine. Base-editor technologies use the same sgRNAs as the traditional Cas9 endonuclease approach. The deamination reactions lead to one or more DNA mismatches at the target site, which are repaired by the endogenous mismatch repair machinery (bottom). (C) Prime editors consist of a Cas9 nickase fused to a reverse transcriptase (RT), which utilizes the sequence provided in a specialized prime editing guide RNA (pegRNA) to introduce a specific edit at the target site. The pegRNA contains the spacer and scaffold sequences used in sgRNAs but adds an additional 3′ DNA sequence including an RT template, the desired edit and a primer binding site (PBS) complementary to the nicked strand (top). Upon binding of the PBS to the free end of the nicked DNA strand, the fused reverse transcriptase incorporates the edit and any additional homologous sequence encoded in the RT template into the nicked DNA strand (middle). Upon DNA re-annealing, a 5′ flap is present, which is cleaved by endogenous exonucleases. The edit is then copied to the opposite strand by mismatch repair machinery or during DNA replication (bottom). A, adenine; APOBEC1, apolipoprotein B mRNA editing enzyme, catalytic polypeptide 1; C, cytosine; Cas9, CRISPR-associated protein 9; G, guanine; I, inosine; U, uracil; T, thymine.

The emergence of CRISPR technologies has revolutionized biomedical research (Doudna and Charpentier, 2014). The initial iteration used the Cas9 nuclease and guide RNA to introduce site-specific double-strand DNA (dsDNA) breaks that are repeatedly repaired by non-homologous end joining (NHEJ) before an error occurs, resulting in a short insertion/deletion (indel) at the target site (Fig. 2A) (Brinkman et al., 2018). In coding regions, this indel can cause a frameshift leading to the creation of downstream premature stop codons and consequent nonsense-mediated decay of the transcript or impaired translation into protein. However, unlike RNAi silencing approaches, it can also be used to probe regulatory elements enriched in non-coding variants through the use of multiple guides to generate deletions across such regions (Diao et al., 2017). As an alternative approach to probe regulatory regions, investigators engineered fusion proteins composed of an endonuclease-dead Cas9 (dCas9) fused to transcriptional repressors (CRISPRi) (Gilbert et al., 2013) or activators (CRISPRa) (Gilbert et al., 2014). New versions of the tools (CRISPRoff and CRISPRon) allow for more stable repression or activation of targeted regulatory elements, at least when regulatory elements can be targeted by introducing or removing DNA methylation marks (Amabile et al., 2016; Nuñez et al., 2021).

The CRISPR/Cas9 system was quickly adapted to facilitate homology-directed repair (HDR), allowing for the precise introduction (or repair) of mutations at target loci (Fig. 1A) (Cong et al., 2013; Mali et al., 2013). For the first time, investigators could more readily introduce precise mutations in non-coding or coding elements and assess the effect on gene regulation and cellular phenotype (Ajore et al., 2022). Cas9-mediated HDR is also amenable to high-throughput screening (Findlay et al., 2014, 2018); however, competition between HDR and NHEJ repair pathways leads to complex readouts, complicating the downstream interpretation of these results. Next-generation CRISPR technologies have helped solve this problem by bypassing the need for repair of dsDNA breaks.

Base editing and prime editing are two novel approaches for precision gene editing that avoid creating dsDNA breaks, significantly improving the ratio of precision edits to random indels (Anzalone et al., 2019; Gaudelli et al., 2017; Komor et al., 2016). Base editors come in two categories: cytosine base editors (CBEs), which promote the conversion of a C:G to a T:A base pair, and adenine base editors (ABEs), which convert an A:T into a G:C base pair. Structurally, base editors consist of a mutant Cas9 capable of introducing single-strand cuts (Cas9 nickase) or that has no cleavage activity fused to a deaminase enzyme (APOBEC1 in the case of CBEs and TadA in the case of ABEs) (Fig. 2B). Because the deaminase activity of the Cas9 fusion protein is dictated by proximity to the target nucleotide (A or C), a specific set of positions within the guide sequence are ‘editable’ and combinatorial editing can occur, occasionally leading to the introduction of multiple non-synonymous mutations in the same allele.

Prime editors consist of a Cas9 nickase fused to a reverse transcriptase domain, utilizing the template provided on a modified guide RNA to introduce specific DNA changes at the target site (Fig. 1C) (Anzalone et al., 2019). Although the rate of NHEJ is slightly higher than with base editors, the ability to introduce specific mutations without restrictions with respect to position within the guide sequence represents a major advantage of this technology. The technology has recently been adapted to facilitate the creation of large desired deletions (Choi et al., 2022) and insertions (Anzalone et al., 2022), and it is likely that new versions will emerge with improved on-target efficiency and lower rates of NHEJ, similar to updated versions of base editors (Koblan et al., 2018).

Endogenous gene targeting has traditionally been limited due to the low efficiency of homologous recombination. A solution emerged with the discovery of RNA silencing, which has been used to study the effect of putative genes driving Diamond-Blackfan anemia (Box 1) (Ebert et al., 2005), the regulation of fetal hemoglobin (Box 1) (Sankaran et al., 2008), and variant effects from GWASs on clotting disorders (de Vries et al., 2019), red blood cell traits (Nandakumar et al., 2019) and immune dysregulation (Box 1) (Peters et al., 2017).

The CRISPR/Cas9 system (Fig. 2) represents a more reliable and stable version of RNA silencing and has become the gold standard for validating the effect of variants on blood cell phenotypes through targeted gene knockout (Anderson et al., 2019; Giani et al., 2016), disruption of *cis*-regulatory elements (Guo et al., 2017) or the precise introduction of variants using homology-directed repair (HDR) (Wienert et al., 2017). The clinical applications of the technology are perhaps even more exciting. CRISPR/Cas9-based therapies are currently being tested for the amelioration of blood disorders in a rapidly growing number of clinical trials (Table 1) (Kanter et al., 2021), and a few are close to garnering U.S. Food and Drug Administration (FDA) approval, including exa-cel, which uses CRISPR/Cas9 to disrupt the erythroid-specific B-cell lymphoma/leukemia 11A (BCL11A; Box 1) enhancer and thus reactivate the production of fetal hemoglobin in autologous CD34^+^ HSPCs. This particular approach is a promising treatment for sickle cell disease (Box 1) and beta-thalassemia (Frangoul et al., 2021).

**Table 1.**
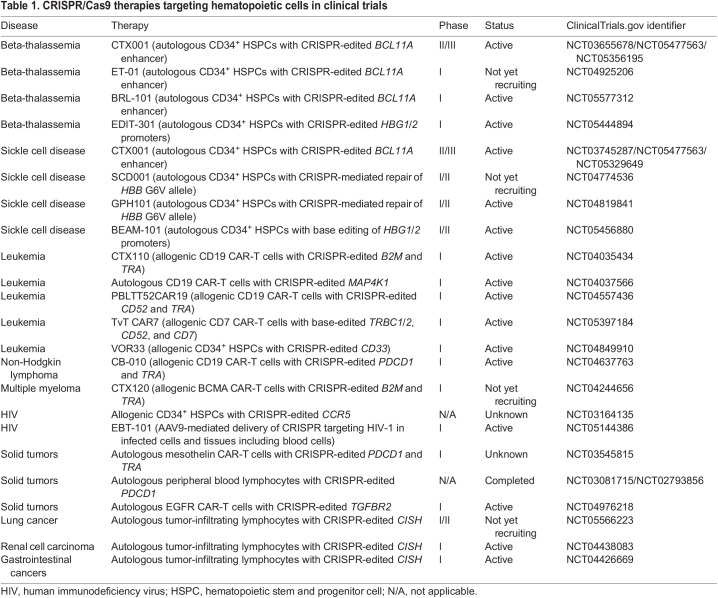
CRISPR/Cas9 therapies targeting hematopoietic cells in clinical trials

Although CRISPR allows for the precise introduction of individual variants at endogenous loci, to overcome the variant-to-function bottleneck, investigators needed a way to edit thousands of putative sites in a single experiment. Fortunately, CRISPR is amenable to multiplexing, allowing investigators to probe multiple regulatory elements in parallel, such as BCL11A enhancers or the *cis*-regulatory elements in the *HBS1L-MYB* intergenic region, both associated with fetal hemoglobin and other red blood cell phenotypes (Canver et al., 2015, 2017). However, this approach is imprecise, relying on the random incorporation of small insertions/deletions at the site of a putative variant or the generation of large deletions surrounding the variant using a paired guide approach. Multiple second-generation CRISPR tools have been developed that help circumvent the imprecision of early CRISPR screens. CRISPRa and CRISPRi allow for site-specific recruitment of active or repressive epigenetic machinery, respectively, allowing researchers to probe thousands of putative regulatory elements associated with blood cell traits in a single experiment (Morris et al., 2021 preprint; Nasser et al., 2021). Base editing has also proven to be an effective tool for massively parallel mutation scanning, enabling validation of GWAS hits (Cuella-Martin et al., 2021; Hanna et al., 2021) and saturation mutagenesis of individual genes (Lue et al., 2023; Sangree et al., 2022). And, similar to CRISPR/Cas9, base editors have already entered the clinic, with two clinical trials for sickle cell disease and leukemia actively recruiting patients (Kingwell, 2022). Finally, prime editing, which represents the most precise endonuclease-mediated repair process to date, has been successfully applied to primary human hematopoietic cells (Petri et al., 2022), used for saturation editing (Erwood et al., 2022) and will surely be used to screen GWAS hits in the near future.

“As we develop methods for targeting HSPCs in their native environment through *in vivo* delivery of CRISPR products, the true impact of these discoveries on our understanding and treatment of blood diseases will be felt.”

## Challenges in manipulating the genomes of primary hematopoietic cells

Recent technological advances have facilitated more precise control in manipulating the genomes of mammalian cells. It is only a matter of time before high-throughput base or prime editor screens successfully interrogate the human genome at base-pair resolution. This will undoubtedly be a major step towards solving the variant-to-function problem in genomics. However, most variants uncovered through GWASs are non-coding variants predicted to fall into regulatory elements with tissue-specific and highly restricted activity. Indeed, this may underlie why many expression quantitative trait loci have had limited value for elucidating disease-causal variants identified through GWASs (Mostafavi et al., 2022 preprint). The use of primary hematopoietic cells is ideal in this circumstance, but has historically been limited due to difficulty in maintaining these cells in culture and low transfection efficiencies with considerable toxicity. Fortunately, new protocols for culture of HSPCs have been developed that allow short-term maintenance of stem and progenitor populations, as well as the directed differentiation into various lineages through the addition of cytokine cocktails, allowing investigators to study lineage-specific effects of genetic variation.

HSPCs can now be expanded more than 30-fold during short-term culture through the addition of the small molecules SR1 and UM171 (Boitano et al., 2010; Fares et al., 2014), which are pyrimidoindole derivatives that promote human HSPC self-renewal by altering epigenomic reprogramming (Subramaniam et al., 2020), enabling large-scale screens of individual umbilical cord blood or mobilized peripheral blood samples. If left to their own device, HSPCs maintained *in vitro* will eventually differentiate into mature blood cells with a significant, if not complete, myeloid skew. However, there are protocols for directed differentiation of HSPCs into almost every mature blood/immune cell lineage (Fig. 1).

**Fig. 3.**
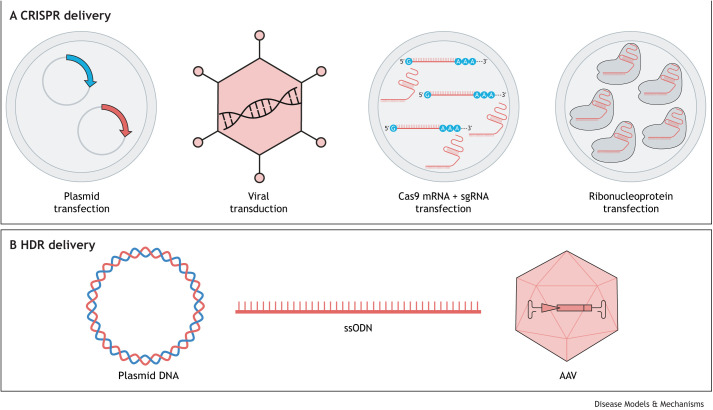
**CRISPR delivery systems.** (A) Approaches for delivery of CRISPR products into hematopoietic cells. (B) Templates utilized for homology-directed repair (HDR). AAV, adeno-associated virus; Cas9, CRISPR-associated protein 9; sgRNA, single-guide RNA; ssODN, single-stranded oligonucleotide.

With the ability to expand human HSPCs and differentiate them into almost any lineage, one would think that hacking hematopoiesis with CRISPR would be a matter of ‘plug and play’. However, it has taken time for CRISPR tools to be optimized for use in primary hematopoietic cells. Early attempts to edit HSPCs using the CRISPR/Cas9 system utilized lentiviral transduction (Heckl et al., 2014) or plasmid DNA transfection (Mandal et al., 2014) (Fig. 3A). These strategies allowed for targeted gene disruption of clinically relevant targets, such as beta-2 microglobin (*B2M*; Box 1) and C**-**C chemokine receptor type 5 (*CCR5*; Box 1). However, high toxicity limited plasmid transfection approaches and low-efficiency knockout was observed with lentiviral approaches, possibly due to HSPC intolerance to constitutive expression of Cas9. These complications prevented high-efficiency gene disruption that could have permitted phenotypes to be screened on the bulk population of cells. An alternative strategy emerged, driven by success in mouse embryo knockout experiments, whereby the raw components of CRISPR [Cas9 protein and the single-guide RNA (sgRNA)] were directly delivered into HSPCs through electroporation (Gundry et al., 2016; Hendel et al., 2015) (Fig. 3A). This approach has proven effective for studying the phenotypic effect of gene disruption in HSPCs (Nakauchi et al., 2022), as well as for therapeutic gene editing of hematopoietic cells, including all of the clinical trials that use CRISPR to edit HSPCs in patients with beta-thalassemia and sickle cell disease (Frangoul et al., 2021) (Table 1). Importantly, the approach does not require phenotypic readout in HSPCs populations, as investigators have edited HSPCs and differentiated them into erythroid (Wu et al., 2019) or neutrophil populations (Rao et al., 2021) prior to exploring a phenotype. The delivery of templates for HDR into HSPCs has also been fine-tuned (Fig. 3B). Initial attempts to deliver plasmids or single-stranded oligodeoxynucleotides (ssODNs) were successful, but demonstrated low-efficiency knock-in. More recently, investigators have found that adeno-associated virus (AAV)-mediated delivery of HDR templates leads to higher knock-in efficiencies and an improved ratio of HDR to non-homologous end joining (NHEJ) (Romero et al., 2019). However, there remains some controversy as to whether long-term HSCs are more effectively repaired by ssODN or AAV templates, as *in vitro* and *in vivo* data have not always correlated (Pattabhi et al., 2019).

**Figure:**
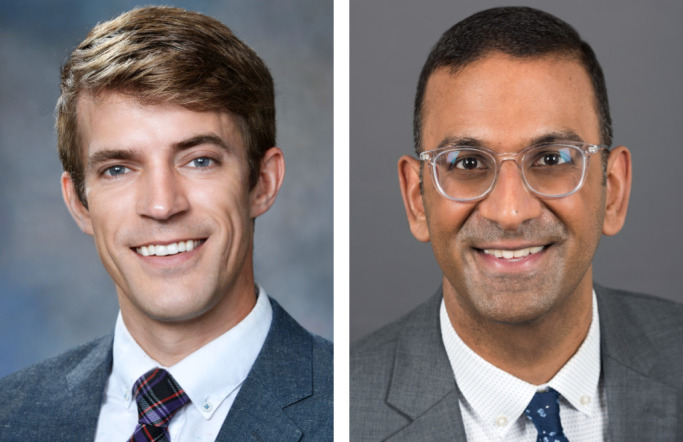
Michael Gundry (left) and Vijay G. Sankaran (right)

The final step in optimizing CRISPR or newer generation genome-editing tools for use in HSPCs is ongoing, with a number of groups currently working on developing protocols for high-content CRISPR or other editing screens in primary cells and with potential single-cell readouts (Bock et al., 2022). Nevertheless, the impact of efficient gene-editing of human HSPCs on the field of hematopoiesis has already been enormous. In a short timeframe, we have seen multiple instances in which these technologies have been used to model or explore disease pathogenesis and the same strategy used to model the disease has become the potential cure (Table 1). As we develop methods for targeting HSPCs in their native environment through *in vivo* delivery of CRISPR products, the true impact of these discoveries on our understanding and treatment of blood diseases will be felt.

“The continued optimization of novel gene-editing approaches in primary cells will allow for the simultaneous assessment of thousands of variants in a physiologically relevant setting.”

## Concluding remarks and future directions

The continued optimization of novel gene-editing approaches in primary cells will allow for the simultaneous assessment of thousands of variants in a physiologically relevant setting. By combining these techniques with new tools for cellular barcoding (Ludwig et al., 2019; Sankaran et al., 2022), we can better characterize the clonal dynamics of native hematopoiesis and discover the mechanisms through which germline or somatically acquired mutations perturb this complex and dynamic process (Qiu et al., 2022). For instance, single-cell omics and clonal tracking were recently used to study how a common somatically acquired mutation found in clonal hematopoiesis perturbs early myeloid progenitor states through alterations in CpG methylation (Nam et al., 2022).

It remains to be seen how effective this approach will be for modeling polygenic traits/disease with subtle *in vitro* phenotypes. Many hematopoietic alterations, such as clonal hematopoiesis, take decades to manifest into a clinical phenotype, such as the onset of a blood cancer, if at all (Fabre et al., 2022; Robertson et al., 2022). It is unclear whether phenotypic changes will declare themselves at early time points. The use of xenotransplantation models, whereby edited human hematopoietic stem or progenitor cells are transplanted into immunocompromised mice, can technically be used to maintain edited cells for longer intervals, but studies have shown that a small fraction of transplanted human HSPCs engraft in these mice, at least in earlier models (Cheung et al., 2013; Sharma et al., 2021). This limits the ability to screen large numbers of variants, but may be improved with new models that enable higher levels of engraftment with more diverse cell types (Cosgun et al., 2014; Martinov et al., 2021; McIntosh et al., 2015; Sargent et al., 2022; Song et al., 2021). Alternatively, screening for molecular phenotypes using assays such as Perturb-seq (Adamson et al., 2016) may uncover perturbed gene regulatory networks within mutant clones that have not yet manifested into obvious cellular phenotypes.

During the past 50 years, we have deciphered the human genome and developed tools for precise manipulation of its individual elements. The next 50 years will likely be defined by applying our knowledge of the genetic basis of disease through personalized pharmacogenomics and therapeutic genome editing. As we work to comprehensively define the contribution of common and rare genetic variation to blood and immune diseases, we should aim to keep the translational implications of our work front and center. The genomic era in medicine has arrived.
